# Loss of Cubilin, the intrinsic factor-vitamin B12 receptor, impairs visceral endoderm endocytosis and endodermal patterning in the mouse

**DOI:** 10.1038/s41598-019-46559-0

**Published:** 2019-07-15

**Authors:** Aitana Perea-Gomez, Olivier Cases, Vincent Lelièvre, Maria V. Pulina, Jérôme Collignon, Anna-Katerina Hadjantonakis, Renata Kozyraki

**Affiliations:** 1 Institut Jacques Monod, Université de Paris, CNRS, Paris, F-75013 France; 2 Centre de Recherche des Cordeliers, INSERM, UMRS-1138, Université de Paris, Paris, F-75006 France; 3 CNRS UPR 3212, INCI, 5 rue Blaise Pascal, F-67084 Strasbourg, France; 4 Developmental Biology Program, Sloan Kettering Institute, Memorial Sloan Kettering Cancer Center, New York, USA; 5 Present Address: Université Côte d’Azur, CNRS, Inserm, iBV France; 6 Present Address: The Rockefeller University, New York, USA

**Keywords:** Embryology, Gastrulation

## Abstract

The visceral endoderm is a polarized epithelial monolayer necessary for early embryonic development in rodents. A key feature of this epithelium is an active endocytosis and degradation of maternal nutrients, in addition to being the source of various signaling molecules or inhibitors required for the differentiation and patterning of adjacent embryonic tissues. Endocytosis across the visceral endoderm epithelium involves specific cell surface receptors and an extensive sub-membrane vesicular system with numerous apical vacuoles/lysosomes. We previously reported that Cubilin, the endocytic receptor for intrinsic factor-vitamin B12, albumin and apolipoproteinA-I/HDL allows maternal nutrient uptake by the visceral endoderm. In the present study, we show that the germline ablation of Cubilin impairs endodermal and mesodermal patterning, and results in developmental arrest at gastrulation. Notably, visceral endoderm dispersal is impeded in Cubilin null embryos. We further confirm the essential role of Cubilin in nutrient internalization by the early visceral endoderm and highlight its involvement in the formation of apical vacuoles. Our results reveal essential roles for Cubilin in early embryonic development, and suggest that in addition to its nutritive function, Cubilin sustains signaling pathways involved in embryonic differentiation and patterning.

## Introduction

Three and a half days after fertilization (E3.5) the mouse blastocyst consists of an outside layer of polarized trophectodermal cells and a cluster of inner cells, the inner cell mass (ICM). One day later the ICM contains two lineages, the epiblast and the primitive endoderm (PrE), a unicellular epithelial layer beneath the epiblast^1,2^. After the blastocyst implants into the maternal endometrium, both cell types of the ICM differentiate. The PrE differentiates to form the parietal endoderm that underlies the mural trophectoderm, and an epithelial monolayer, the visceral endoderm (VE), that surrounds the entire embryo. The epiblast matures and from E6.5, when gastrulation begins, gives rise to the mesoderm, the definitive endoderm and the ectoderm^3,4^. The VE will generate the visceral yolk sac (VYS) and contribute to the endoderm of the gut^5–7^. The VE and its derivative the VYS have key roles in fetal nutrition and homeostasis during development. In addition, the VE provides signals necessary for epiblast patterning including the positioning of the primitive streak (PS) at the posterior side of the embryo, and the formation of the anterior neuroectoderm^8–10^.

Cubilin (Cubn, ~460 kDa) is a multiligand receptor predominantly expressed in the embryonic and adult gut and kidney, as well as in the VYS^11^. Cubilin consists of an N-terminal stretch followed by 8 epidermal growth-factor (EGF) like repeats and 27 CUB (Complement C1r/C1s, Uegf, BMP1) domains involved in ligand binding. It is a peripheral membrane protein with no transmembrane domain or GPI anchor. Cubilin forms complexes with at least two other proteins: Amnionless (Amn) and Lrp2/Megalin, both single-spanning transmembrane proteins involved in Cubilin trafficking and/or internalization^11–15^.

Cubilin has site-dependent functions. In the gut it is critical for the physiological uptake of vitamin B12 complexed with its carrier, the gastric intrinsic factor. In the kidney, Cubilin is a key protein required for tubular protein reabsorption, including of albumin (reviewed in^16,17^). In the VYS, Cubilin is essential for maternal lipid and protein uptake and inhibition of its function impairs normal embryonic growth^18–21^. In the embryo-proper, Cubilin acts as an accessory receptor for Fgf8 and is required for cephalic neural crest cell migration and head morphogenesis^22^.

Mutations in human *CUBN* (10p12.33-p13) result in the rare pediatric Immerslund-Gräsbeck syndrome characterized by megaloblastic anemia, neurological findings and frequent proteinuria^23–25^. Recently, missense variants in *CUBN* were also associated with nephrotic syndrome and albuminuria, colorectal cancer progression and a risk of neural tube defects^26–28^.

We and others previously showed that in the VYS Cubilin interacts with both Amn and Lrp2^18,29^. We also reported that Lrp2 inactivation affects the internalization of Cubilin ligands and leads to functional Cubilin deficiency in the renal epithelium^18,30,31^. Nevertheless the expression of Lrp2 in the VYS is dispensable for embryonic growth indicating that in this site Lrp2 is not absolutely required, including for nutrient uptake^32^. By contrast, the genetic ablation of the second Cubilin partner *Amn* results in defective PS assembly and impaired apical distribution of Cubilin in the VYS^13,29,33^. Full *Cubn* deletion is also embryonic lethal and associated with both defective VYS function and mesodermal defects^21^. However, the molecular and cellular nature of these embryonic and extraembryonic defects has never been investigated, and the role of Cubilin in the early VE remains an open question.

To gain further insight into the early embryonic functions of Cubilin, we generated *Cubn* null mice using the previously reported *Cubn* floxed allele^22,30^ and the widespread deletor PGK-Cre line. We show that the lack of *Cubn* impairs gastrulation and leads to major endodermal and mesodermal defects. Our data reveal that Cubilin is an early VE marker whose absence results in a failure of VE dispersal resulting in defective gut endoderm morphogenesis at gastrulation, as well as impaired endocytic function.

## Results

### Cubilin expression in the primitive endoderm and its derivatives between E3.5 and E8.5

**Figure 1:**
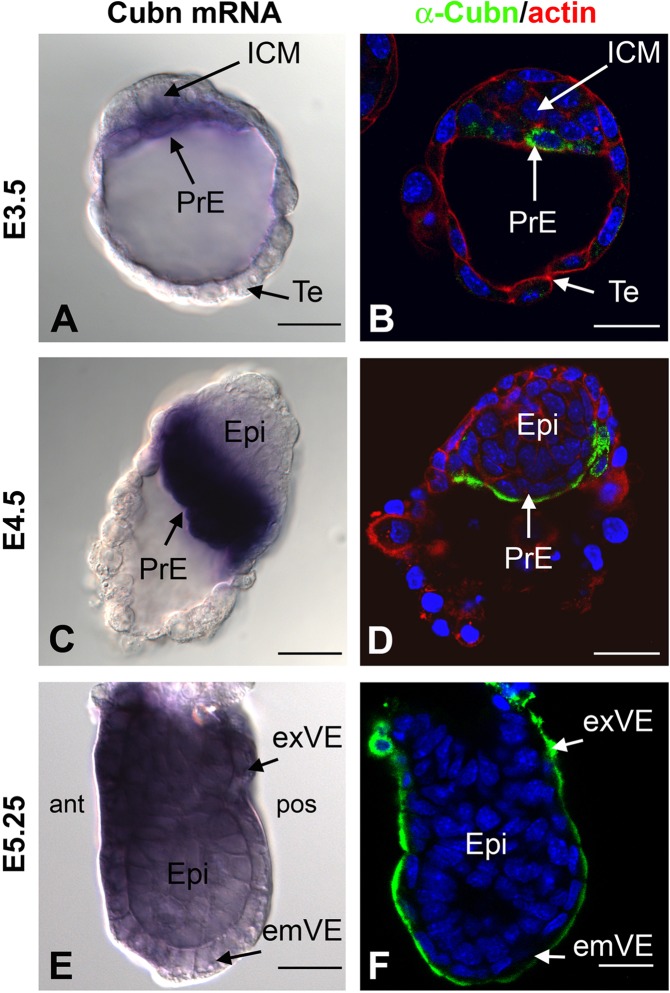
Expression of *Cubn* mRNA and protein prior to gastrulation. (**A**,**C** and **E**) Whole mount *in situ* hybridization of *Cubn* mRNA (**A**) at embryonic day 3.5 (E3.5) showing *Cubn* expression at the level of the primitive endoderm (PrE). (**C**) At the late blastocyst stage E4.5, strong *Cubn* mRNA expression is found in the PrE. (**E**) At the early postimplantation stage E5.25, *Cubn* expression is enriched in the extraembryonic visceral endoderm (exVE). (**B**,**D** and **F**) Whole mount immunofluorescence detects Cubilin (in green) in the cytoplasm of the PrE (**B**) and subapical region of the PrE cells. (**D**) Phalloidin is in red; nuclei colored by Hoechst are shown in blue. (**F**) At E5.25, Cubilin protein is detected both in the extraembryonic and embryonic subapical region of the VE cells. ant: anterior, ICM: inner cell mass, emVE: embryonic visceral endoderm, Epi: epiblast, post: posterior, Te: trophectoderm. Scale Bars: 20 μm in (**A**,**B**) 25 μm in (**C**,**D**) 40 μm in (**E**,**F**).

**Figure 2:**
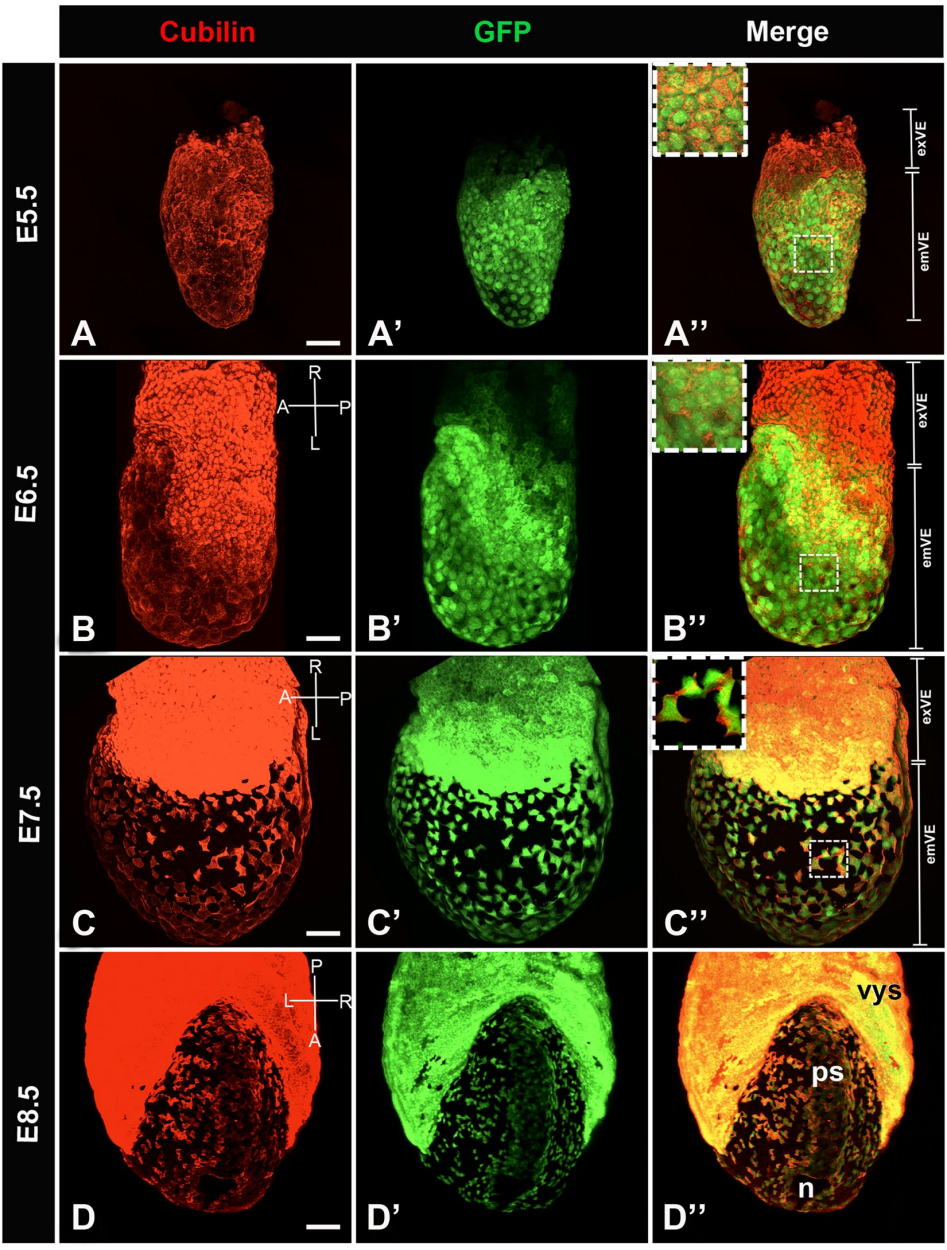
Distribution of Cubilin protein in primitive endoderm cellular derivatives from postimplantation E5.5 to E8.5. Whole mount image of immunofluorescence for Cubilin (red) in *Afp-*GFP VE reporter embryo (green). (**A–A”**) At E5.5, Cubilin is expressed by VE cells (**A**) while GFP is detected exclusively in the emVE (**A’**). All GFP positive cells express Cubilin. (**A”**) Inset shows Cubilin expression in emVE cells. (**B–B”**) By E6.5, at the onset of gastrulation, Cubilin is strongly expressed by exVE cells. (**B**) GFP expression spread more proximally into the exVE (**B’**). All GFP positive cells express Cubilin (**B”**) in emVE. (**C–C”**) At E7.5, late bud/early head fold stage, Cubilin is expressed throughout the exVE and in dispersed emVE cells. (**C**) All GFP cells express Cubilin (**C’**,**C”**). Inset shows Cubilin expression in squamous emVE cells. (**D–D’**) At E8.5 (5–6 somites stage), Cubilin is expressed strongly in the visceral yolk sac (vys), and the dispersed emVE cells. (**D**) Cubilin expression domain matches integrally with this of GFP (**D’**,**D”**). emVE: embryonic visceral endoderm, exVE: extraembryonic visceral endoderm, n: node, ps: primitive streak. Scale bars: 20 μm.

To detail early Cubilin expression we combined whole-mount *in situ* hybridization and fluorescent whole-mount immunostaining. In E3.5 blastocysts *Cubn* mRNA and protein are detected in ICM cells adjacent to the blastocoelic surface, the final position of PrE cells. (Fig. 1A,B). In these cells Cubilin is not localized at the plasma membrane but is distributed around the nucleus in a punctate pattern (Fig. 1B). Additionally, at the same stage Cubilin immunostaining reveals a scattered intracellular and membrane distribution in the trophectodermal layer, as previously reported (Fig. 1B and ref.^18^). Around one day later corresponding to E4.5, PrE cells start to migrate along the trophectoderm as they form the parietal endoderm (Fig. 1C,D). At this stage *Cubn* mRNA is abundantly expressed in the PrE and Cubilin immunostaining is clearly associated with the apical plasma membrane reflecting the polarization of PrE cells (Fig. 1C,D). Between E5.25 and E5.5, the PrE cells that remain in contact with the epiblast and the extraembryonic ectoderm form the embryonic (em) and extraembryonic (ex) VE respectively (Fig. 1E,F). From this stage onward the *Cubn mRNA* signal appears to progressively decline in the emVE (Suppl. Fig. 1 and ref.^34^). In sharp contrast, the protein signal is readily detected throughout the VE (Figs 1F and 2). The reason for this discrepancy is unclear but may be due to the perdurance of the protein.

Around E5.5, emVE cells begin to express alpha-fetoprotein (AFP), a typical VE marker^35^. To clearly define the distribution of Cubilin in the emVE at this stage we used the transgenic mouse line *Afp-GFP*^36^ where GFP is expressed under the regulatory sequences of *Afp* providing a useful tool to visualize VE and its derivatives. Confocal imaging of whole embryos shows the expression of Cubilin in all GFP-positive cells (Fig. 2A–A”).

From E6.5 onward (Fig. 2B–D”), Cubilin is expressed with GFP in the emVE and progressively the exVE. At E7.5, the emVE cells are dispersed by the definitive endoderm precursors (Fig. 2C’)^5^. As shown in Fig. 2C–D”, Cubilin marks all dispersed GFP-positive emVE cells thus behaving as a bona-fide emVE marker.

### *Cubn* is required for morphogenesis during gastrulation

**Figure 3:**
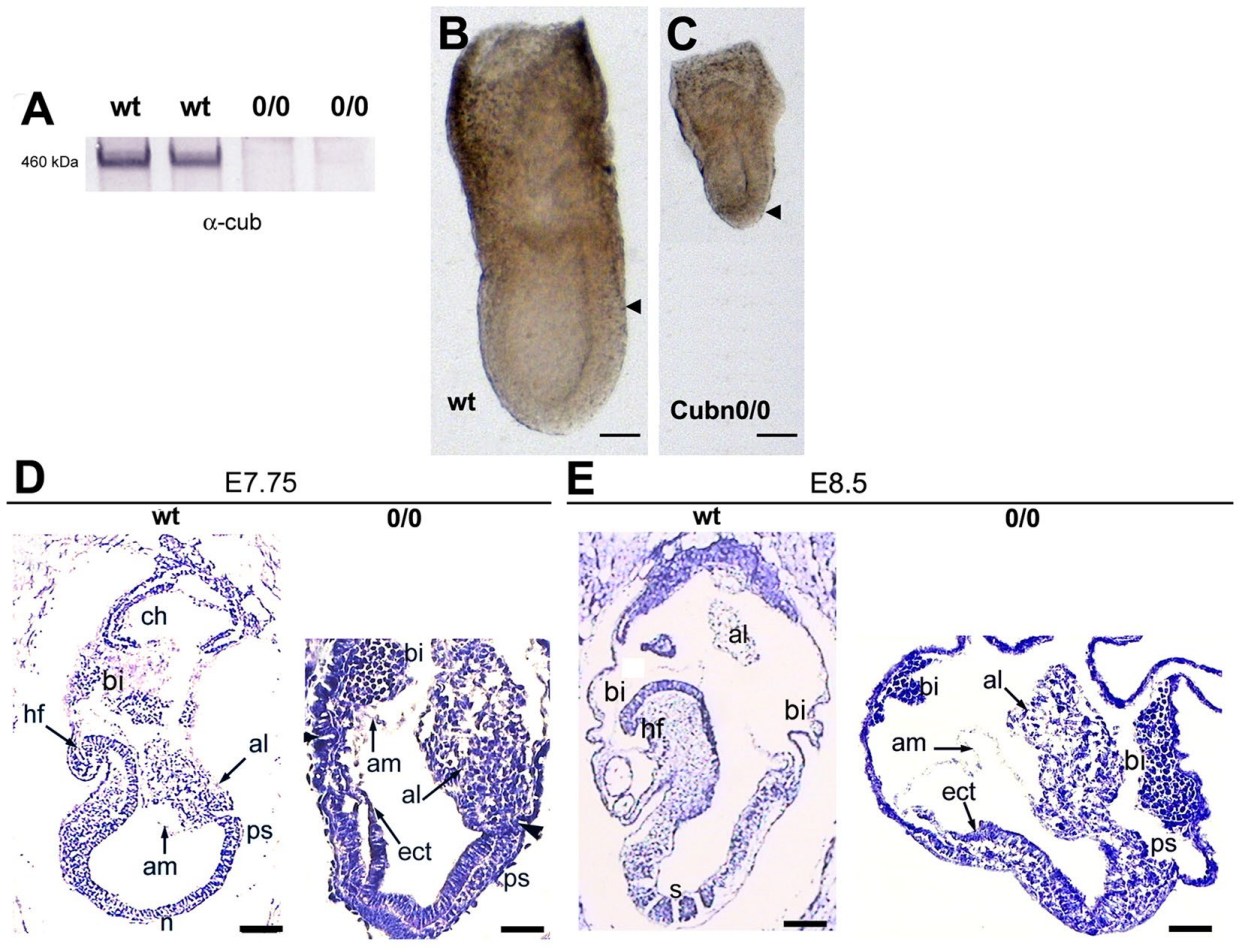
Embryonic and extraembryonic defects in *Cubn*^*0*/*0*^ embryos. (**A**) Cubilin protein is absent from embryonic extracts of *Cubn*^*0*/*0*^ embryos at E7.5. (**B**,**C**) At E7.5 both embryonic and extraembryonic portions are formed in the mutants but the embryonic region is small compared to the extraembryonic part. Arrowheads indicate the demarcation between extraembryonic and embryonic VE; anterior is to the left. (**D**) At E7.75 the mutant embryos have no obvious head mesenchyme; the amnion (am) is formed, the allantois (al) is short, and abnormally thick blood islands (bi) are associated with the visceral yolk sac. (**E**) At E8.5 no somites (s) are formed in the mutants; anterior is to the left. al: allantois, am: amnion, bi: blood island, ch: chorion, ect: ectoderm, hf: headfold, n: node, ps: primitive streak, s: somite. Scale bars: 80 μm in (**B**,**C**) 50 μm in (**D**,**E**).

To obtain complete *Cubn* inactivation, *Cubn Lox*/+ mice^22,30^ were first crossed with a line ubiquitously expressing *Cre* under the control of the *PGK* promoter, active during oogenesis^37^, to generate a *Cubn* null allele named *Cubn*^*0*^. Intercrosses of *Cubn*^*0*/+^ animals then gave rise to *Cubn*^*0*/*0*^ embryos where no residual expression of Cubilin was observed (Fig. 3A). *Cubn*^*0*/*0*^ embryos develop normally until the onset of gastrulation at E6.5 (data not shown and Suppl Fig. 2A–C’). At E7.5 they are smaller than their control littermates and have a shorter PS (Fig. 3B,C and ref.^21^). Additionally, in the mutants the proximally-located extra-embryonic region of the conceptus appears more developed than the distal embryonic region (Fig. 3B,C). Histological analysis confirms that gastrulation had occurred in E7.75 *Cubn*^*0*/*0*^ embryos as evidenced by the presence of mesoderm cells (Fig. 3D). However, unlike the wild-type or heterozygous littermate (control) the mutants do not show head-folds or foregut structures (Fig. 3D). At E8.5, while in control embryos the first somites are detectable and the heart starts to form, in the mutant embryos the embryonic region remains small and disorganized without any signs of somites or other trunk structures (Fig. 3E). The extraembryonic tissues, including exVE, amnion and allantois are formed, but the allantois remains short and large, and never attaches to the chorion (Fig. 3E). Abnormal enlarged blood islands are seen in the mutant VYS (Fig. 3E and ref.^21^). These observations are consistent with a previous report on Cubilin loss of function^21^ and indicate that Cubilin is required for proper patterning and morphogenesis of the gastrulating embryo.

### Morphological and molecular defects of the *Cubn*-deficient VE

**Figure 4:**
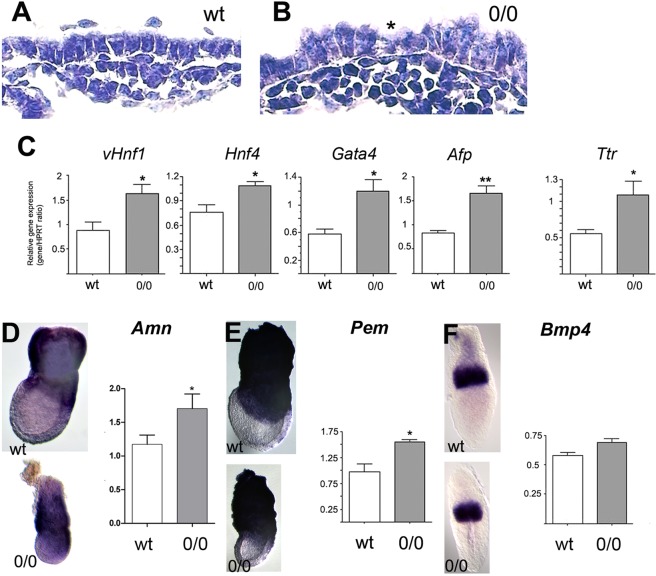
Visceral endoderm defects in *Cubn*^*0*/*0*^ embryos. (**A**,**B**) The extraembryonic VE (exVE) cells are abnormally tall in E7.5 mutants; cuboidal (asterisk) and tall columnar cells are intermixed. (**C**) Quantitative RT-PCR analysis of VE differentiation markers at E7.5; *vHnf1*, *Hnf4*, *Gata4*, *a-foetoprotein* (*Afp*) and *transthyretin* (*Ttr*). (**D**,**E**) WMHIS (E7.5) and quantitative RT-PCR analysis (E7.5) for the expression of *Amnionless* (**D**) and *Pem* (**E**) two markers of exVE. (**F**) WMHIS (E6.5) and quantitative RT-PCR analysis for the expression of *Bmp4*, a marker of the extraembryonic ectoderm and mesoderm. Three E7.5 *Cubn*^*0*/*0*^ and control littermates are included in the quantitative RT-PCR analysis. Scale bars: 100 μm in (**A**,**B**).

The main site of *Cubn* expression during gatrulation is the VE. In *Cubn*^*0*/*0*^ embryos the overall morphology of the VE is altered showing intermixing of tall columnar and more cuboidal cells (Fig. 4A,B). To investigate the molecular defects of the mutant VE, we compared the expression of factors involved in the nutritive and/or hematopoiesis and vasculogenesis functions of the exVE by qRT-PCR and whole mount mRNA *in situ* hybridization analyses. The early (*vHnf1*, *Hnf4*, *Gata4*) and late (*Afp* and *transthyretin*) VE differentiation markers and the exVE markers *Amn* and *Pem* are expressed in mutant embryos, indicating that VE differentiation and molecular regionalization occur in the absence of Cubilin function (Fig. 4C–E). However, the expression of VE markers appears significantly increased in E7.5 mutant embryos compared to control littermates (Fig. 4C). During gastrulation, VE cells in the embryonic region are dispersed and displaced proximally towards the extraembryonic region as definitive endoderm is formed and progressively covers the distal embryonic region of the conceptus. The increased expression of VE markers in *Cubn* mutants as compared to controls might be linked to the relative small size of the embryonic region in these mutants, developmental delay and/or to defects in the generation of the definitive endoderm germ layer.

Despite these endodermal defects, the expression of *Bmp4*, a known inducer of visceral endoderm involved in the proximodistal patterning of the embryo^38^, is not altered in the extra-embryonic ectoderm of the mutants (Fig. 4F).

### Mesodermal and endodermal defects in the *Cubn* null mutants

**Figure 5:**
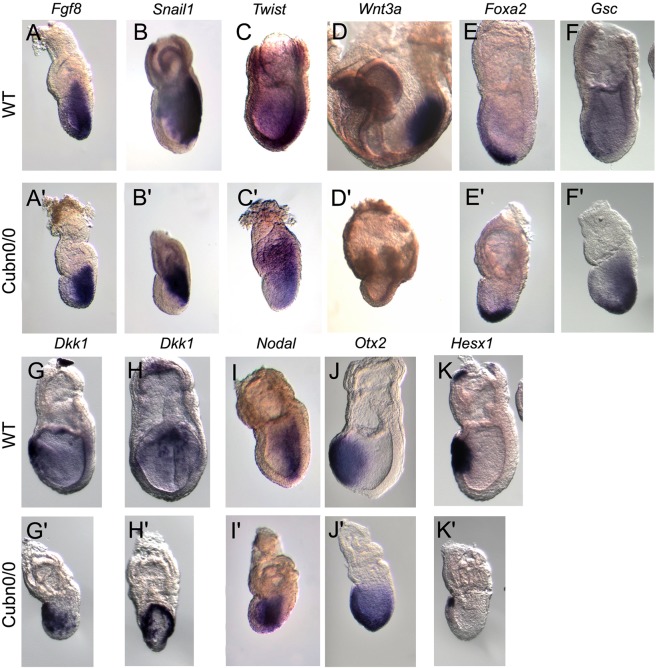
Marker analysis of *Cubn*^*0/0*^ mutants between E7.5 and E8.5. At least three mutants were analyzed for each marker by WMISH. (**A**,**A’**) As compared with control littermates *Cubn*^*0*/*0*^ embryos express homogeneous and strong levels of *Fgf8* in the primitive streak. (**B**,**B’**) *Snail1* is expressed in the streak of the control and the mutants, whereas (**C**,**C’**) the paraxial and presomitic mesoderm marker *Twist* is significantly reduced in *Cubn*^*0*/*0*^ embryos. (**D**,**D’**) The posterior mesoderm marker *Wnt3a* is greatly reduced in E8.5 *Cubn*^*0*/*0*^ embryos. (**E–F’**) The anterior PS and AME markers *Foxa2* and *Gsc* are expressed in the distal most part of the PS. (**G**,**H**) *Dkk1*, a Wnt-signaling inhibitor is expressed in a crescent-shaped domain by anterior definitive endoderm cells. (**G’**,**H’**) In *Cubn*^*0*/*0*^ embryos, the domain of expression expands posteriorly (**G’**) and laterally (**H’**). (**I**,**I’**) Unlike in control littermates *Nodal* is not downregulated in the anterior ectoderm of the mutants. (**J**,**J’**) The anterior neuroectoderm marker *Otx2* is not restricted anteriorly. (**K**,**K’**) The homeobox gene *Hesx1* is expressed in AVE, AME and anterior axial neuroectoderm. In *Cubn*^*0*/*0*^ embryos, *Hesx1* is expressed in a small anterior proximal domain. All panels are lateral views with anterior to the left, except H, H’ showing frontal views of embryos in (**G**,**G’**).

In order to characterize the patterning defects observed in gastrulating *Cubn* null embryos we analyzed the distribution of established mesodermal, endodermal and prospective neuroectodermal markers. Anterior-posterior pattern evidenced by the expression of *Hex or Lefty1* in the anterior visceral endoderm (AVE) and *Brachyury* (*T*) in the nascent mesoderm, is established in *Cubn*^*0*/*0*^ around E6.5 (Suppl. Fig. 2A,B’). However, the expression domain of *T* remains restricted to the proximal most part of the mutant PS (Suppl. Fig. 2A’). Additionally, *Lefty2* has not yet been induced posteriorly (Suppl. Fig. 2B’) and *Nodal*, involved in the formation and maintenance of the PS remains present throughout the epiblast as in control embryos at earlier stages (Suppl. Fig. 2C,C’). We further analyzed *Cubn* null embryos at E7.5. *Fgf8* is required for normal gastrulation and specification of the paraxial mesoderm^39^. In *Cubn* mutants, *Fgf8* expression is upregulated (Suppl. Fig. 2D) and *Fgf8* transcripts are homogenously distributed along the entire PS. This is in contrast to the proximo-distal gradient of *Fgf8* expression observed in E7.5 control littermates (Fig. 5A,A’). *Snail1*, a marker of the embryonic mesoderm is detected in the mutant PS (Fig. 5B,B’ and Suppl. Fig. 2D). These observations indicate that PS and mesoderm formation occur in the absence of Cubilin, however proximo-distal PS extension and/or patterning might be deficient.

In the mouse, the proximal PS produces extra-embryonic mesoderm, the middle streak produces lateral, paraxial and intermediate components of the trunk mesoderm, whereas the anterior-most region of the PS produces the anterior mesendoderm (AME), the axial mesoderm and definitive endoderm. The expression of the paraxial and presomitic marker *Twist* is significantly reduced and mainly marks the mutant extra-embryonic mesoderm as well as mesodermal cells close to the proximal PS in E7.5 *Cubn*^*0*/*0*^ embryos (Fig. 5C,C’ and Suppl. Fig. 2D). One day later, the expression of *Wnt3a*, a WNT ligand essential for caudal somite development^40^ is strongly reduced in the mutants (Fig. 5D,D’). These observations indicate that paraxial mesoderm formation is impaired in Cubilin mutants.

*Foxa2* and *Goosecoid* (*Gsc*), mark anterior PS and AME cells during gastrulation. Both genes are expressed in the mutants although their domains of expression are restricted to the distal part of the PS, and do not extend anteriorly (Fig. 5E–F’). *Dkk1* encodes a *Wnt* antagonist expressed first in the AVE and then in the PS derived AME. In control E7.5 embryos, *Dkk1* transcripts are restricted to the prospective foregut domain in the anterior definitive endoderm. In the mutants, *Dkk1* expression in the outer endoderm layer exhibits a horse-shoe pattern expanding posteriorly around the girth of the embryo (Fig. 5G–H’). This expression is reminiscent of that observed in the VE of E6.5 control embryos. This analysis suggests that AME morphogenesis and definitive endoderm formation are impaired in *Cubn* mutants.

In the ectoderm layer molecular regionalization also takes place during gastrulation with genes like *Nodal* being down-regulated from the anterior epiblast to become restricted to the PS region, and others like *Otx2* being down-regulated from the posterior epiblast to be maintained in the prospective anterior neurectoderm. In *Cubn* mutants, the expression of both *Nodal* and *Otx2* is abnormally maintained in the entire epiblast (Fig. 5I–J’ and Suppl. Fig. 2D). The expression of *Hesx1*, a marker of AVE, AME, and anterior neurectoderm, is strongly reduced in the mutants (Fig. 5K,K’).

Together our results indicate that paraxial mesoderm and anterior mesendoderm formation are defective in *Cubn* mutants, which may in turn affect ectoderm regionalization.

### Visceral endoderm dispersal is deficient in *Cubn* null embryos

**Figure 6:**
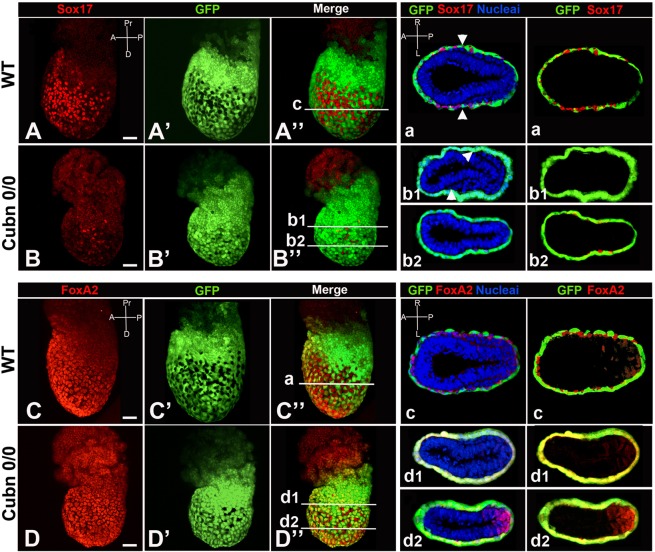
Cubilin inactivation disrupts embryonic visceral endoderm (emVE) dispersal. Whole mount image of immunofluorescence for Sox17 (red) in *Afp-GFP* VE reporter embryo (green). (**A–A”**) By E7.25 egressing definitive endoderm (DE) cells robustly express Sox17 (Sox17^high^) but lack GFP expression. (a) Cross section showing Sox17^high^GFP^−^ DE cells at the surface of the embryo. Arrowheads mark the leading edge of the mesodermal wings. (**B–B”**) In *Cubn*^*0*/*0*^ embryos, transgenic for the *Afp-GFP* VE reporter the number of Sox17^high^GFP^−^ DE cells is greatly diminished. (b1,b2) Cross sections show a quasi absence of egressing DE cells. Whole mount image of immunofluorescence for Foxa2 (red) and *Afp-GFP* VE reporter embryo (green). (**C–C”**) By E7.25 Foxa2 is expressed in emVE and DE precursors. (c) Cross section showing intermixed Foxa2 + GFP^−^ and Foxa2 + GFP^+^ cells. Low levels of Foxa2 are also found in the primitive streak (PS) or in cells leaving the PS. (**D–D”**) In *Cubn* mutants, the Foxa2 + GFP^+^ cell cohort (emVE) is predominant. (d1,d2) Cross sections confirming the abundance of Foxa2 + GFP^+^ cells at the surface of the embryo. Foxa2-positive cells are observed at the level of the PS. Scale bars: 20 μm.

We next investigated whether the above gastrulation defects originating at the PS impaired the dispersal of emVE cells known to take place at these stages^6^. The ingression of endoderm progenitors through the distal PS is necessary for definitive endoderm (DE) formation. Between E7.0 and E7.5 epiblast-derived cells intermingle with and disperse the emVE cells without displacing them to the extraembryonic regions^5^. To further examine the role of Cubilin in DE formation and concomitant emVE cell dispersal, we used live imaging and fluorescent cell labeling. The distribution of the definitive endoderm progenitor markers Sox17 and Foxa2^6,41^ was analyzed in *Cubn*^*0/0*^-*Afp-GFP* transgenic embryos around E7.25, a time when emVE dispersal is extensive (Fig. 6). Sox17 is required for endoderm specification and is expressed in the DE prior to and after intercalation with the emVE. As previously reported Sox17 is strongly expressed in egressing DE progenitors and at lower levels in the emVE (Fig. 6A–A”)^6^. Sox17-positive DE precursors are readily identified in surface renderings and transverse epiblast sections either surrounded by GFP-positive emVE cells or traveling alongside the mesoderm (Fig. 6A–A”,a). In *Cubn* mutants most of the cells on the surface epithelium express low levels of Sox17 (Fig. 6B–B”,b). A strong Sox17 signal is observed in some isolated cells (Fig. 6B), and the distribution of GFP is almost uniform (Fig. 6B’) indicating a failure of emVE dispersal and DE formation. This is confirmed in transverse sections also showing an accumulation of mesodermal cells in the proximal epiblast close to the PS (Fig. 6b1).

Foxa2-positive cell recruitment from the epiblast, transit through the PS and integration/expansion into the endoderm layer is also important for the establishment of the DE^41,42^. Foxa2 is co-expressed with Sox17 showing likewise a low expression levels in emVE cells and high expression levels in DE progenitors^6^. Foxa2-positive cells are either surrounded by GFP-expressing cells, or co-expressed with GFP in emVE (Fig. 6C–C” and c). In the emVE of *Cubn* mutants the distribution of Foxa2 and GFP is generally overlapping (Fig. 6D–D”), suggesting that Foxa2 expressing DE progenitors fail to reach the outer layer and disperse the emVE in these mutants. This is particularly evident in transverse sections across the proximal and distal epiblast of *Cubn* mutants (Fig. 6d1,d2), also showing that the bulk of Foxa2-positive cells accumulate in the mutant PS (Fig. 6d2). Impaired gastrulation and defective emVE dispersal thus appear to be associated, and indeed successive, events in *Cubn* mutants.

### Impaired endocytosis in the *Cubn*-deficient VE

Between E5.0 and E6.5 the VE, and after gastrulation the VYS and its extensive vesicular system are responsible for maternal nutrient uptake and waste exchange between the fetus and its mother. The VE, especially its extraembryonic portion, and the VYS are characterized by the presence of large apical vacuoles/lysosomes in which endogenous and exogenous endocytic markers accumulate, including the lysosome-associated membrane proteins LAMP1/2 and the lysosomal proteinases cathepsins^4,43^. The implication of Cubilin in exogenous/maternal apolipoproteinA-I/HDL endocytosis and degradation, as well as normal blood vessel formation in the VYS was previously reported^18,21^.

**Figure 7:**
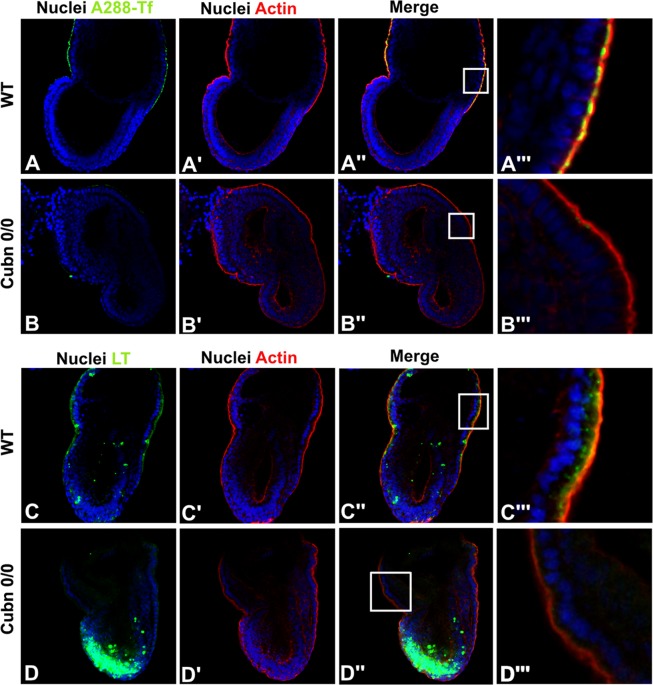
Impaired nutrient endocytosis and lysosomal activity in *Cubn* mutants. (**A–B”’**) Confocal imaging of transferrin uptake (green) and actin staining (red). By E7.5 transferrin uptake is almost exclusively detected in exVE cells. (**A–A”**) Inset of **A”** showing that transferrin accumulates in large structures underneath the plasma membrane (**A”’**). (**B–B”’**) In *Cubn*^*0*/*0*^ embryos, transferrin uptake is abolished. Inset of B” showing the absence of transferrin accumulation (**B”’**). (**C–D”’**) Confocal imaging of LysoTracker (green) and actin staining (red). (**C–C”’**) LysoTracker (LT) staining detects acidification of cellular compartments. By E7.5, some LT-positive punctae are observed in the anterior part of the epiblast (**C–C”**). Inset of **C”** at the level of the exVE cells, LT-positive vesicles indicate the presence of apical vacuoles/lysosomes (**C”’**). (**D–D”**) In *Cubn*^*0*/*0*^ embryos, an enlarged LT-positive domain is observed in the epiblast. (**D”’**) Inset of **D”** in the exVE large LT-positive vesicles are absent, instead small LT-positive vesicles are occasionally detected. All images show single confocal sections, anterior is to the left.

Transferrin is an essential nutrient for the developing embryo, a marker of clathrin-dependent endocytosis and an established Cubilin ligand^31^. We incubated live pre-gastrulating and gastrulating embryos for 5 min at 37 °C with Alexa488-labeled transferrin (A488-Tf). In control E5.5 embryos, A488-Tf accumulates in subapical vesicular structures throughout the VE (Suppl. Fig. 3A,B). As expected, in E6.5 and E7.5 embryos, the A488-Tf signal appears more pronounced in the exVE compared to the emVE (Suppl. Fig. 3C and Fig. 7A–A”’). In *Cubn* null embryos, no signal is observed, further confirming the crucial role of Cubilin in Tf uptake by the VE (Fig. 7B–B”’).

Deficient nutrient uptake may affect cell proliferation and/or cell survival. Cell proliferation identified by the M-phase cell marker phospho-histone H3 (H3S28P) is only marginally reduced in *Cubn* deficient embryos (Suppl. Fig. 4A–D). In contrast, apoptosis followed by TUNEL staining is much higher in the mutant embryos, suggesting that an active cell death process may account for the smaller size of the mutant epiblast (Suppl. Fig. 4A,B,D).

Lysotracker is a product that freely permeates cell membranes and accumulates in intracellular compartments with low pH; namely endolysosomal compartments and/or autophagolysosomal structures^44,45^. Control and mutant embryos were labeled for 15 min or 30 min at 37 °C with LysoTracker Green (LT) and then incubated for 15 more minutes in the absence of the tracer (the chase period). At the end of the chase period, a strong vesicular staining, presumably of the apical vacuoles is seen in the exVE of control embryos (Fig. 7C–C”’) (n = 3). Additionally, a scarce punctuated staining of the emVE and the control epiblast is evident (Fig. 7C,C”). In *Cubn* deficient embryos (n = 5) the LT signal is undetectable in VE cells suggesting defective formation of the acidic compartments (Fig. 7D–D”’). In contrast, an intense signal is observed in the anterior epiblast (Fig. 7D,D”), most likely reflecting increased cell death^46^ also identified by the TUNEL staining.

**Figure 8:**
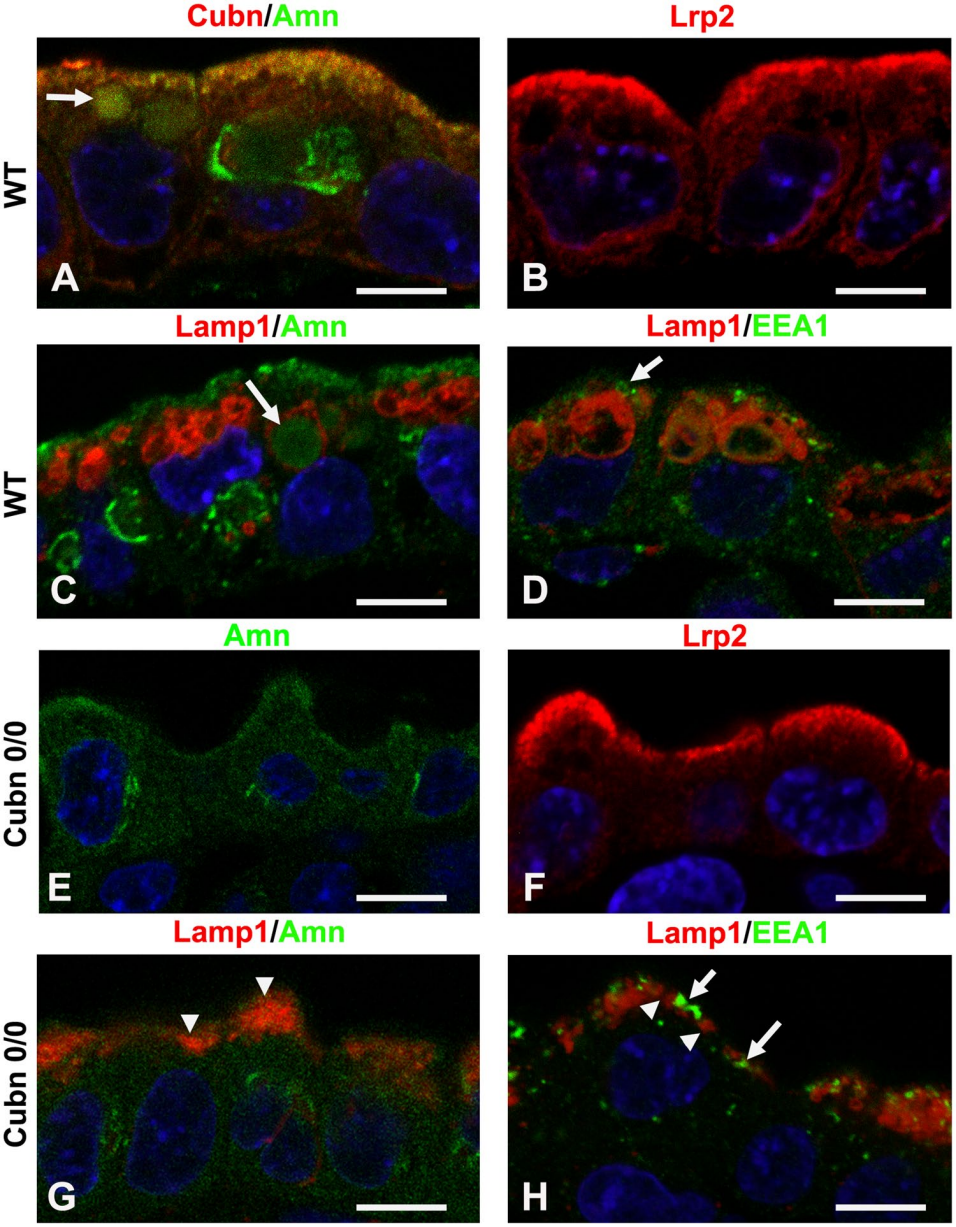
Altered expression of endocytic markers in the exVE of *Cubn* mutants. (**A**) Cubilin (red) and Amn (green) colocalize at the plasma membrane. A faint Amn signal is also observed in the large subapical vesicles (arrow). (**B**) Lrp2 is mainly localized at the plasma membrane. (**C**) LAMP1 marks lysosomes that display large vacuolar morphologies in the exVE cells. The faint vesicular Amn immunoreactivity is found in LAMP1-positives vesicles (arrow). (**D**) EEA1 is a marker of endosomal processing and decorates small disc-shaped structures underneath the apical plasma membrane (arrow) that are distinct from LAMP1 vesicles. (**E**) In *Cubn*^*0*/*0*^ embryos, Amn displays an abnormal dispersion in the cytoplasm whereas (**F**) Lrp2 staining remains apical. (**G**) In *Cubn*^*0*/*0*^ embryos, LAMP1- or Amn-positive large vacuoles are absent. LAMP1- positive smaller and denser vacuoles are observed underneath the plasma membrane (arrowheads). (**H**) In *Cubn*^*0*/*0*^ embryos, both EEA1 and LAMP1 vesicles are localized underneath the apical plasma membrane. EEA1 decorates small dense vesicles (arrows) distinct from the small LAMP1-positive vesicles (arrowheads). Scale bars: 15 μm.

Immunomorphological analysis of control and *Cubn* deficient embryos (Fig. 8) shows that Cubilin, Amn and Lrp2 are expressed and/or co-localize at the apical pole of exVE cells (Fig. 8A,B). Additionally, Amn can also be found in large apical vacuoles positive for the lysosomal marker Lamp1 (Fig. 8C). In control embryos the EEA1 expressing endosomes are disc-shaped, of various sizes and are localized closer to the apical plasma membrane than the much larger Lamp1- positive apical vacuoles (Fig. 8D).

In the mutants, Amn loses its polarized distribution and is diffusely expressed throughout the VE cell (Fig. 8E). By contrast, Lrp2 is readily found at the apical pole of the exVE cells (Fig. 8F). The Lamp1-positive structures are smaller, fewer and localized just beneath the apical membrane together with sparse, small and condensed EEA1 vesicles (Fig. 8G,H). Furthermore, the endosomal marker clathrin adaptor complex 1 (AP-1) is exclusively seen in small subapical vesicles and the expression of Lamp2, a marker of the apical vacuoles/lysosomes is barely detectable in exVE extracts (Suppl. Fig. 5A,B). Collectively these results show defective endosome and lysosome formation in *Cubn* mutants and strongly support a role for Cubilin in endo-lysosomal assembly and/or function in the VE.

## Discussion

We have investigated the embryonic and cellular processes that require Cubilin function during gastrulation. *Cubn* deficiency results in mesodermal, endodermal and ectodermal patterning defects in gastrulation. Furthermore, we show that Cubilin is an early pan-VE marker involved in emVE dispersal, as well as the formation of the VE endocytic apparatus. In keeping with previous findings in other contexts, we establish the importance of Cubilin in VE nutrient endocytosis^18,20,21,47^.

As previously reported^21^, loss of *Cubn* is not compatible with embryonic survival. *Cubn* null embryos initiate gastrulation, but do not undergo normal organogenesis and display various mesodermal, endodermal and ectodermal defects. Here we show that anterior-posterior patterning is established and that PS formation and mesoderm migration are initiated in *Cubn* mutants. However, subsequent PS elongation and patterning are impaired and paraxial mesoderm population is reduced. Additionally, the AME, which is instrumental for the induction and maintenance of anterior neuroectoderm identity and the inhibition of Wnt signaling is formed, but the domains of expression of the AME-specific genes *Foxa2* and *Gsc* are abnormal, as the anterior mesendoderm does not seem to extend anteriorly. Finally, the dispersal of emVE is impaired in *Cubn* mutants and the ingression of DE precursors is restricted to the posterior epiblast.

Within the VE, Cubilin is co-expressed with the classical VE marker AFP^36,48,49^. It is worth noticing however that whereas Cubilin protein is readily detected throughout the VE, the *Cubn mRNA* becomes downregulated in the emVE around E6.5. Although this difference may be due to the lower sensitivity of the *in situ* hybridization technique, it could alternatively reveal a rapid downregulation of the *Cubn mRNA* in the emVE, as previously reported for other VE markers concomitant, with protein perdurance in a population which is not actively dividing^5,50^. In the VE Cubilin is also co-expressed with Lrp2 and Amn. *Amn* in the extra-embryonic tissues is essential for normal gastrulation and its mutant displays molecular defects similar to those observed in *Cubn* mutants^13^. Although the lack of Lrp2 in the VE does not affect embryonic development, it is possible that the persistence of the three partners is necessary for an optimal Cubilin, Amn and Lrp2 activity in the VE. Supporting this hypothesis loss of apical expression of Lrp2 in the VE is thought to contribute to the gastrulation defects of the *Mesd* and *Dab2* mutants^51,52^.

Inactivation of *Cubn* exclusively in the epiblast does not interfere with mesoderm formation or the induction of anterior neuroectodermal characters^22^. It is therefore likely that the phenotype of *Cubn* null mutants at E7.5 is due to the function of Cubilin in the VE. During mouse gastrulation, the VE is an essential component of the embryo-maternal interface required for the exchange of nutrients and waste. *Cubn* null mutants show defective gastrulation, developmental arrest, increased apoptosis in the epiblast and defective endocytosis of nutrients. These observations indicate that the nutritional function of the VE is severely impaired in *Cubn* mutants. Cross-interactions between VE and the underlying tissues, the extra-embryonic ectoderm and the epiblast, are also essential for embryo patterning as exemplified by the role of VE secreted Nodal antagonists in the restriction of PS inducing signals to the posterior epiblast^9^. Whether the failure to restrict Nodal expression in *Cubn* mutants is solely explained by the developmental arrest of these embryos or is also the result of impaired signaling between VE and the adjacent epiblast will require further investigations.

Degradative endocytosis of nutrients and signaling molecules is a key function of the VE. During the early steps of the process extra-cellular molecules bound to cell surface receptors are transported to subapical early endosomal vesicles. Mutations that affect these steps lead to developmental defects^52–55^. The later stages of endocytosis involve the engulfment of the endosomal vesicles by the apical vacuoles, specialized lysosomal compartments of the VE^4,56,57^. Lysosomal digestion is essential for nutrient delivery to the embryo-proper and signal termination. Disruption of lysosomal formation thus results in gastrulation defects^58–60^. Inactivation of the late endosomal adaptor p18, abundantly expressed in the VE, causes a gastrulation phenotype similar to that of *Cubn* mutants^60^. It is of interest that in the *p18* null embryos, the normally large apical vacuoles/lysosomes are not detectable; only small lysosomal structures are evident, and Cubilin as well as Lrp2 are predominantly found within mutant VE cells. The authors propose that the disorganization of the endosomal/lysosomal compartments affects the trafficking and apical insertion of these membrane receptors^60^. Defective membrane expression of Cubilin, Lrp2 and most likely Amn may therefore contribute to the abrogation of nutrient uptake and subsequently affect epiblast viability.

Increased cell death in the epiblast, as the one we report here, was also observed in embryos lacking *mVam2*, a late endosomal protein, involved in BMP signaling^59^. In these mutants no large apical vacuoles are assembled and the delivery of internalized proteins, such as albumin or transferrin, from the endosomes to lysosomes is deficient despite normal initial uptake^59^. In the *Cubn* mutant both initial transferrin uptake and formation of apical vacuoles appear to be affected. No transferrin accumulates in subapical vesicles and the EEA1-positive endosomes are small, sparse and locate just beneath the plasma membrane. Furthermore, the morphology and topology of the apical vacuoles are severely perturbed as suggested by the Lysotracker and Lamp1 staining. Thus, both the initial, and to a greater extent the late, steps of endocytosis appear to be affected by the loss of Cubilin.

How Cubilin, a peripheral membrane protein might impair endocytosis is not clear. Either the formation of the Cubilin-Amn-Lrp2 macromolecular complex is an essential step for early endocytosis in the VE or additional Cubilin partners may be involved.

In summary, the present study uncovers important functions of murine Cubilin during the peri-gastrulation period. Cubilin deficiency affects epiblast patterning and VE morphogenesis. In view of the requirement of Cubilin for the proper assembly and/or maintenance of the apical vacuoles residing within the VE epithelium, we propose that Cubilin-mediated endocytosis in the VE is necessary for both nutrition and morphogenetic signaling in the early embryo.

## Materials and Methods

### Ethics statement

Animal procedures were conducted in compliance with approved institutional protocols (INSERM and comité d’éthique en experimentation animale Charles Darwin No. 5, Permit Number 01519.01, Memorial Sloan Kettering Cancer Center (MSKCC), Institutional Animal Care and Use Committee (IACUC) under Protocol No. 03-12-017) and in accordance with the provisions for animal care and use described in the European Communities council directive of 22 September 2010 (2010/63/EU). Deep anesthesia for terminal procedures was provided with a ketamine/xylazine cocktail (80 mg/10 mg/kg).

### Mice

All mice were maintained under pathogen-free conditions, according to institutional guidelines. The conditional targeting vector for *Cubn*-deficient mice was described elsewhere^30^. *Cubn* null (*Cubn*^*0*/*0*^) embryos were genotyped as described^30^. *Afp-*GFP^TG/+^ were genotyped as described^5^. Gestation (E0.0) was considered to have begun at midnight before the morning when a copulation plug was found. A small fragment of the extra-embryonic region of E6.5 and E7.5 embryos or a piece of yolk sac of older embryos was used for genotyping according to standard procedures.

### Immunocytochemistry, histology and *in situ* hybridization

Mouse embryos were staged according to their morphology. Whole-mount *in situ* hybridization was performed as described previously^9^. Sense and antisense RNA probes were transcribed from the appropriate promoters using T3, SP6 and T7 RNA polymerase to obtain digoxigenin riboprobes (Roche Diagnostics, Meylan, France). *Immunohistochemistry*: Dissected embryos were fixed for one hour in 4% parafolmadehyde in PBS, equilibrated in 10% sucrose overnight, embedded in OCT (Tissue-Tek, Miles) and sectioned in alternate sagittal, frontal or transverse sections at 10 μm using a cryostat Leica CM1900. Whole embryos or sections were incubated for 2 hours at room temperature or overnight at 4 °C with primary antibodies; rabbit anti-gamma adaptin 1 (AP1G1, Abcam, ab22051), rabbit anti-Cubilin^30^, rabbit anti-Foxa2 (Abcam, ab40874), rabbit anti-Lamp1 (Abcam, ab24170), sheep anti-Lrp2^30^, goat anti-amnionless (Santa-Cruz, K14, sc-46726), goat anti-EEA1 (Santa-Cruz, N19, sc-6415), goat anti-Sox17 (R&D Systems, AF1924). Secondary antibodies used were Alexa 488- or Cy3-conjugated donkey anti-rabbit (Jackson Immunoresearch Laboratories, West Grove, PA; 1:500), Alexa 488- or Cy3-conjugated donkey anti-mouse (Invitrogen, Eugene, OR; 1:500), and Alexa 488- or Cy3-conjugated donkey anti-goat (1:500). Nuclear staining was achieved by 20 min incubation at room temperature in Hoechst 33342 (Invitrogen, Eugene, OR). Cortical actin was revealed by incubation overnight at 4 °C with Alexa 647-Phalloidin (Invitrogen, Eugene, OR). Images were collected by confocal microscopy (Leica SP5 AOBS and Zeiss LSM 710) and processed using ImageJ software. *Histology*: Sections were stained with 0.1% cresyl violet.

### Real time PCR

Poly-A RNA from single embryos was isolated using Dynabeads mRNA DIRECT kit (Invitrogen, Cergy-Pontoise, France). Reverse transcription of RNA (600 ng/sample) was performed using iScript select cDNA synthesis kit (BioRad, Hercules, CA) according to the manufacturer’s protocol. Real-time PCR was performed using a BioRad iCycler and IQ SYBR Green Supermix (BioRad), reactions were performed in triplicate. Transcript levels were calculated using the standard curves generated using serial dilutions of cDNA obtained by reverse transcription of control RNA samples then normalized to HPRT. Primer sequences for the indicated genes are included in Supplementary Table 1. The graphs plot the mean ± s.d. of the fold expression of the control and mutant littermates used. The number of specimens is indicated in the figure legends section. *T*-test statistical analysis showed significant differences at ^(**)^p < 0.01, and ^(*)^p < 0.1. Amplification specificities were assessed by melting curve analyses and amplicons were sequenced.

### Lyso tracker staining of mouse embryos

Embryos were dissected free of decidua and extra-embryonic membranes discarded. LysoTracker green DND-26 (ThermoFisher) was prepared at 5 mM in Hanks BBS. Embryos were incubated at 37 °C for 15 min or 30 min with similar results, and then incubated for 15 min in the absence of the tracer (chase). After washing, embryos were fixed in 4% paraformaldehyde overnight. Actin staining was performed by incubating embryos in Phalloidin (Alexa Fluor Phalloidin 647, ThermoFisher) overnight. Images were collected by confocal microscopy (Zeiss LSM 710) and processed using ImageJ software.

### Transferrin uptake

Embryos were removed from decidua and extra-embryonic membranes discarded. Transferrin alexa Fluor 488 (ThermoFisher) was prepared at 25 μg/ml in DMEM. Embryos were incubated at 37 °C for 5 min. After washing, embryos were fixed in 4% paraformaldehyde overnight. Actin staining was performed by incubating embryos in Phalloidin (Alexa Fluor Phalloidin 647, ThermoFisher) overnight. Images were collected by confocal microscopy (Zeiss LSM 710) and processed using ImageJ software.

### TUNEL staining and cell proliferation

The ApopTag Red *In Situ* Apoptosis Detection Kit (Merck Millipore, S7165) was used to detect apoptotic cells. For cell proliferation, anti-phospho-histone H3 (H3S28P; 1:250, Merck Millipore) followed by Alexa 488-conjugated goat anti-rabbit (1:200, Invitrogen) was used. Nuclear staining was achieved by a 20-min incubation in Hoechst 33342 (Invitrogen). Images were collected by confocal microscopy (8 μm/section; LSM710 ConfoCor 3, Carl Zeiss) and processed using ImageJ software. Total numbers of TUNEL- and H3S28P-positive profiles were counted in 7 consecutive sections. TUNEL- and H3S28P-positive profiles were automatically detected using the following set of parameters in Volocity Image Analysis Software (Perkin-Elmer): (1) Find Objects by Intensity; (2) exclude objects smaller than 7 μm^2^; (3) separate touching objects greater than 25 μm^2^. The apoptotic index (TUNEL) and mitotic index (H3S28P) were calculated as the percentage of cells positive for each marker to the total number of Hoechst 33342 -positive (nuclei marker, blue) cells in ectoderm (epiblast and extraembryonic ectoderm) and embryonic visceral endoderm per embryo. All graphs were generated using GraphPad Prism version 7 and data are shown as mean and SEM. Mann-Whitney U test was used for analysing cell number; TUNEL and H3S28P profiles and ***p < 0.001 was considered highly significant.

### Western blot

For western blot analysis (30 μg/sample) embryonic extracts were used. Embryos were lysed in a PBS buffer (10 mM NaH_2_PO_4_, 150 mM Nacl, 6 mM CaCl_2_) with 1% Triton X-100 (Merck), 1 mM sodium orthovanadate, and Complete mini EDTA-free protease inhibitor cocktail tablets (Roche Diagnostics), pH 7.4. Immunoblotting analyses were performed by standard procedures using ECL reagents as described by the manufacturer (GE Healthcare). To standardize the protein expression across samples, we used an anti-beta-actin goat antibody at a dilution of 1/5,000 (Abcam, ab-8229) and a rabbit anti-GAPDH at a dilution of 1/1000 (Cell Signaling Technology, 5174). Primary antibodies used were rabbit anti-cubilin^30^ and anti-Lamp2 (ThermoFisher, PA1-655).

## Supplementary information

Supplementary-Info

## References

[1] Bassalert C, Valverde-Estrella L, Chazaud C (2018). Primitive Endoderm Differentiation: From Specification to Epithelialization. Curr. Top. Dev. Biol..

[2] Rossant J, Tam PPL (2009). Blastocyst lineage formation, early embryonic asymmetries and axis patterning in the mouse. Dev. Camb. Engl..

[3] Beddington RS, Robertson EJ (1998). Anterior patterning in mouse. Trends Genet. TIG.

[4] Bielinska M, Narita N, Wilson DB (1999). Distinct roles for visceral endoderm during embryonic mouse development. Int. J. Dev. Biol..

[5] Kwon GS, Viotti M, Hadjantonakis A-K (2008). The endoderm of the mouse embryo arises by dynamic widespread intercalation of embryonic and extraembryonic lineages. Dev. Cell.

[6] Viotti M, Nowotschin S, Hadjantonakis A-K (2014). SOX17 links gut endoderm morphogenesis and germ layer segregation. Nat. Cell Biol..

[7] Nowotschin Sonja, Setty Manu, Kuo Ying-Yi, Liu Vincent, Garg Vidur, Sharma Roshan, Simon Claire S., Saiz Nestor, Gardner Rui, Boutet Stéphane C., Church Deanna M., Hoodless Pamela A., Hadjantonakis Anna-Katerina, Pe’er Dana (2019). The emergent landscape of the mouse gut endoderm at single-cell resolution. Nature.

[8] Martinez-Barbera JP, Beddington RS (2001). Getting your head around Hex and Hesx1: forebrain formation in mouse. Int. J. Dev. Biol..

[9] Perea-Gomez A (2002). Nodal antagonists in the anterior visceral endoderm prevent the formation of multiple primitive streaks. Dev. Cell.

[10] Stuckey DW, Di Gregorio A, Clements M, Rodriguez TA (2011). Correct patterning of the primitive streak requires the anterior visceral endoderm. PloS One.

[11] Moestrup SK (1998). The intrinsic factor-vitamin B12 receptor and target of teratogenic antibodies is a megalin-binding peripheral membrane protein with homology to developmental proteins. J. Biol. Chem..

[12] Coudroy G (2005). Contribution of cubilin and amnionless to processing and membrane targeting of cubilin-amnionless complex. J. Am. Soc. Nephrol. JASN.

[13] Tomihara-Newberger C (1998). The amn gene product is required in extraembryonic tissues for the generation of middle primitive streak derivatives. Dev. Biol..

[14] Udagawa T (2018). Amnionless-mediated glycosylation is crucial for cell surface targeting of cubilin in renal and intestinal cells. Sci. Rep..

[15] Yagi S, Shiojiri N (2017). Identification of novel genetic markers for mouse yolk sac cells by using microarray analyses. Placenta.

[16] Eshbach ML, Weisz OA (2017). Receptor-Mediated Endocytosis in the Proximal Tubule. Annu. Rev. Physiol..

[17] Kozyraki, R. & Cases, O. Cubilin, the intrinsic factor-vitamin B12 receptor in development and disease. *Curr*. *Med*. *Chem*., 10.2174/0929867325666181008143945 (2018). 10.2174/0929867325666181008143945 30295181

[18] Assémat E (2005). Expression and role of cubilin in the internalization of nutrients during the peri-implantation development of the rodent embryo. Biol. Reprod..

[19] Assémat E (2005). Overlapping expression patterns of the multiligand endocytic receptors cubilin and megalin in the CNS, sensory organs and developing epithelia of the rodent embryo. Gene Expr. Patterns GEP.

[20] Sahali D (1988). Characterization of a 280-kD protein restricted to the coated pits of the renal brush border and the epithelial cells of the yolk sac. Teratogenic effect of the specific monoclonal antibodies. J. Exp. Med..

[21] Smith BT (2006). Targeted disruption of cubilin reveals essential developmental roles in the structure and function of endoderm and in somite formation. BMC Dev. Biol..

[22] Cases O (2013). Cubilin, a high affinity receptor for fibroblast growth factor 8, is required for cell survival in the developing vertebrate head. J. Biol. Chem..

[23] Aminoff M (1999). Mutations in CUBN, encoding the intrinsic factor-vitamin B12 receptor, cubilin, cause hereditary megaloblastic anaemia 1. Nat. Genet..

[24] Grasbeck R, Gordin R, Kantero I, Kuhlback B (1960). Selective vitamin B12 malabsorption and proteinuria in young people. A syndrome. Acta Med. Scand..

[25] Kozyraki R (1999). The intrinsic factor-vitamin B12 receptor, cubilin, is a high-affinity apolipoprotein A-I receptor facilitating endocytosis of high-density lipoprotein. Nat. Med..

[26] Al-Tassan NA (2015). A new GWAS and meta-analysis with 1000Genomes imputation identifies novel risk variants for colorectal cancer. Sci. Rep..

[27] Pangilinan F (2012). Evaluation of common genetic variants in 82 candidate genes as risk factors for neural tube defects. BMC Med. Genet..

[28] Reznichenko A (2012). CUBN as a novel locus for end-stage renal disease: insights from renal transplantation. PloS One.

[29] Strope S, Rivi R, Metzger T, Manova K, Lacy E (2004). Mouse amnionless, which is required for primitive streak assembly, mediates cell-surface localization and endocytic function of cubilin on visceral endoderm and kidney proximal tubules. Dev. Camb. Engl..

[30] Amsellem S (2010). Cubilin is essential for albumin reabsorption in the renal proximal tubule. J. Am. Soc. Nephrol. JASN.

[31] Kozyraki R (2001). Megalin-dependent cubilin-mediated endocytosis is a major pathway for the apical uptake of transferrin in polarized epithelia. Proc. Natl. Acad. Sci. USA.

[32] Spoelgen R (2005). LRP2/megalin is required for patterning of the ventral telencephalon. Dev. Camb. Engl..

[33] Kalantry S (2001). The amnionless gene, essential for mouse gastrulation, encodes a visceral-endoderm-specific protein with an extracellular cysteine-rich domain. Nat. Genet..

[34] Frankenberg S, Smith L, Greenfield A, Zernicka-Goetz M (2007). Novel gene expression patterns along the proximo-distal axis of the mouse embryo before gastrulation. BMC Dev. Biol..

[35] Dziadek M (1978). Modulation of alphafetoprotein synthesis in the early postimplantation mouse embryo. J. Embryol. Exp. Morphol..

[36] Kwon GS (2006). Tg(Afp-GFP) expression marks primitive and definitive endoderm lineages during mouse development. Dev. Dyn. Off. Publ. Am. Assoc. Anat..

[37] Lallemand Y, Luria V, Haffner-Krausz R, Lonai P (1998). Maternally expressed PGK-Cre transgene as a tool for early and uniform activation of the Cre site-specific recombinase. Transgenic Res..

[38] Zorn AM, Wells JM (2009). Vertebrate endoderm development and organ formation. Annu. Rev. Cell Dev. Biol..

[39] Guo Q, Li JYH (2007). Distinct functions of the major Fgf8 spliceform, Fgf8b, before and during mouse gastrulation. Dev. Camb. Engl..

[40] Takada S (1994). Wnt-3a regulates somite and tailbud formation in the mouse embryo. Genes Dev..

[41] Hallonet M (2002). Maintenance of the specification of the anterior definitive endoderm and forebrain depends on the axial mesendoderm: a study using HNF3beta/Foxa2 conditional mutants. Dev. Biol..

[42] Burtscher I, Lickert H (2009). Foxa2 regulates polarity and epithelialization in the endoderm germ layer of the mouse embryo. Dev. Camb. Engl..

[43] Wada Y (2013). Vacuoles in mammals: a subcellular structure indispensable for early embryogenesis. Bioarchitecture.

[44] Chikte S, Panchal N, Warnes G (2014). Use of LysoTracker dyes: a flow cytometric study of autophagy. Cytom. Part J. Int. Soc. Anal. Cytol..

[45] Maulucci G (2015). Quantitative analysis of autophagic flux by confocal pH-imaging of autophagic intermediates. Autophagy.

[46] Zucker RM (2006). Whole insect and mammalian embryo imaging with confocal microscopy: morphology and apoptosis. Cytom. Part J. Int. Soc. Anal. Cytol..

[47] Le Panse S (1995). Immunofunctional properties of a yolk sac epithelial cell line expressing two proteins gp280 and gp330 of the intermicrovillar area of proximal tubule cells: inhibition of endocytosis by the specific antibodies. Eur. J. Cell Biol..

[48] Dziadek MA, Andrews GK (1983). Tissue specificity of alpha-fetoprotein messenger RNA expression during mouse embryogenesis. EMBO J..

[49] Viotti M, Nowotschin S, Hadjantonakis A-K (2011). Afp::mCherry, a red fluorescent transgenic reporter of the mouse visceral endoderm. Genes. N. Y. N 2000.

[50] Law SW, Dugaiczyk A (1981). Homology between the primary structure of alpha-fetoprotein, deduced from a complete cDNA sequence, and serum albumin. Nature.

[51] Lighthouse JK, Zhang L, Hsieh J-C, Rosenquist T, Holdener BC (2011). MESD is essential for apical localization of megalin/LRP2 in the visceral endoderm. Dev. Dyn. Off. Publ. Am. Assoc. Anat..

[52] Maurer ME, Cooper JA (2005). Endocytosis of megalin by visceral endoderm cells requires the Dab2 adaptor protein. J. Cell Sci..

[53] Schwarz DG, Griffin CT, Schneider EA, Yee D, Magnuson T (2002). Genetic analysis of sorting nexins 1 and 2 reveals a redundant and essential function in mice. Mol. Biol. Cell.

[54] Zheng B (2006). Essential role of RGS-PX1/sorting nexin 13 in mouse development and regulation of endocytosis dynamics. Proc. Natl. Acad. Sci. USA.

[55] Meagher MJ, Braun RE (2001). Requirement for the murine zinc finger protein ZFR in perigastrulation growth and survival. Mol. Cell. Biol..

[56] Batten BE, Haar JL (1979). Fine structural analysis of the effect of trypan blue on the visceral endoderm of the mouse egg cylinder. Acta Anat. (Basel).

[57] Le Panse S, Verroust P, Christensen EI (1997). Internalization and recycling of glycoprotein 280 in BN/MSV yolk sac epithelial cells: a model system of relevance to receptor-mediated endocytosis in the renal proximal tubule. Exp. Nephrol..

[58] Kawamura N (2012). Delivery of endosomes to lysosomes via microautophagy in the visceral endoderm of mouse embryos. Nat. Commun..

[59] Aoyama M (2012). Spatial restriction of bone morphogenetic protein signaling in mouse gastrula through the mVam2-dependent endocytic pathway. Dev. Cell.

[60] Nada S (2009). The novel lipid raft adaptor p18 controls endosome dynamics by anchoring the MEK-ERK pathway to late endosomes. EMBO J..

