# Supplementary material for: Loss of Cubilin, the intrinsic factor-vitamin B12 receptor, impairs visceral endoderm endocytosis and endodermal patterning in the mouse
Source: Sci Rep. 2019 Jul 15;9:10168. doi: 10.1038/s41598-019-46559-0 (PMC6629654; doi:10.1038/s41598-019-46559-0)
Supplement: Supplementary file 1 — Supplementary-Info [file 41598_2019_46559_MOESM1_ESM.docx]

Supplementary information

**Loss of Cubilin, the intrinsic factor-vitamin B12 receptor, impairs visceral endoderm endocytosis and endodermal patterning in the mouse**

Aitana Perea-Gomez^1§^, Olivier Cases^2^, Vincent Lelièvre^3^, Maria V. Pulina^4#^, Jérôme Collignon^1^, Anna-Katerina Hadjantonakis^4^, Renata Kozyraki^2*^


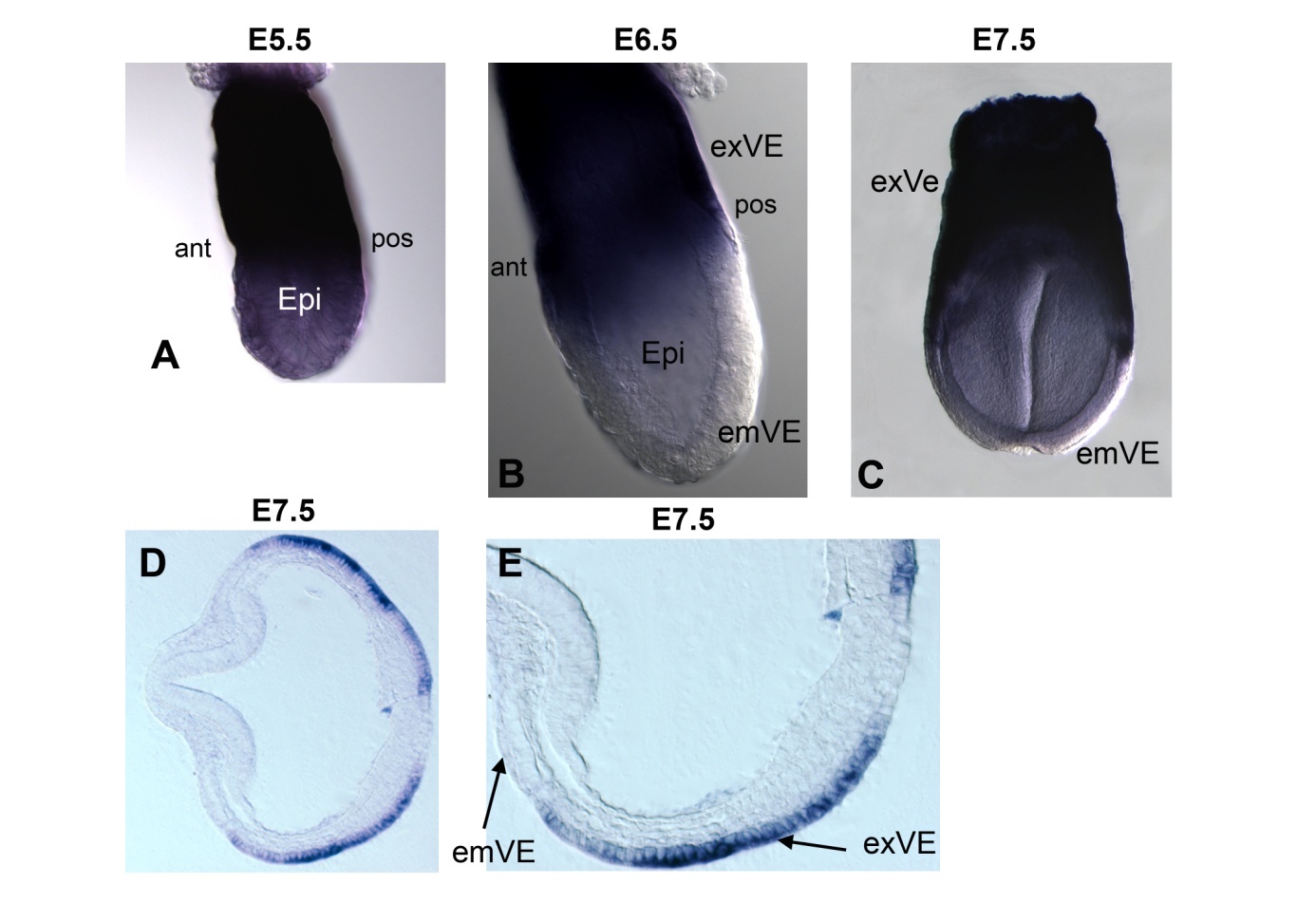


**Suppl. Figure 1**: Expression of *Cubn* mRNA between E5.5 and E7.5. WMHIS of *Cubn* mRNA showing strong expression at the level of the extraembryonic visceral endoderm (exVE) at E5.5 (**A**), E6.5 gastrulation stage (**B**) and E7.5 (**C**). (**D**, **E**) Cross section through a E7.5 embryo showing *Cubn* expression in exVE cells. ant: anterior, emVE: embryonic visceral endoderm, Epi: epiblast, pos: posterior.


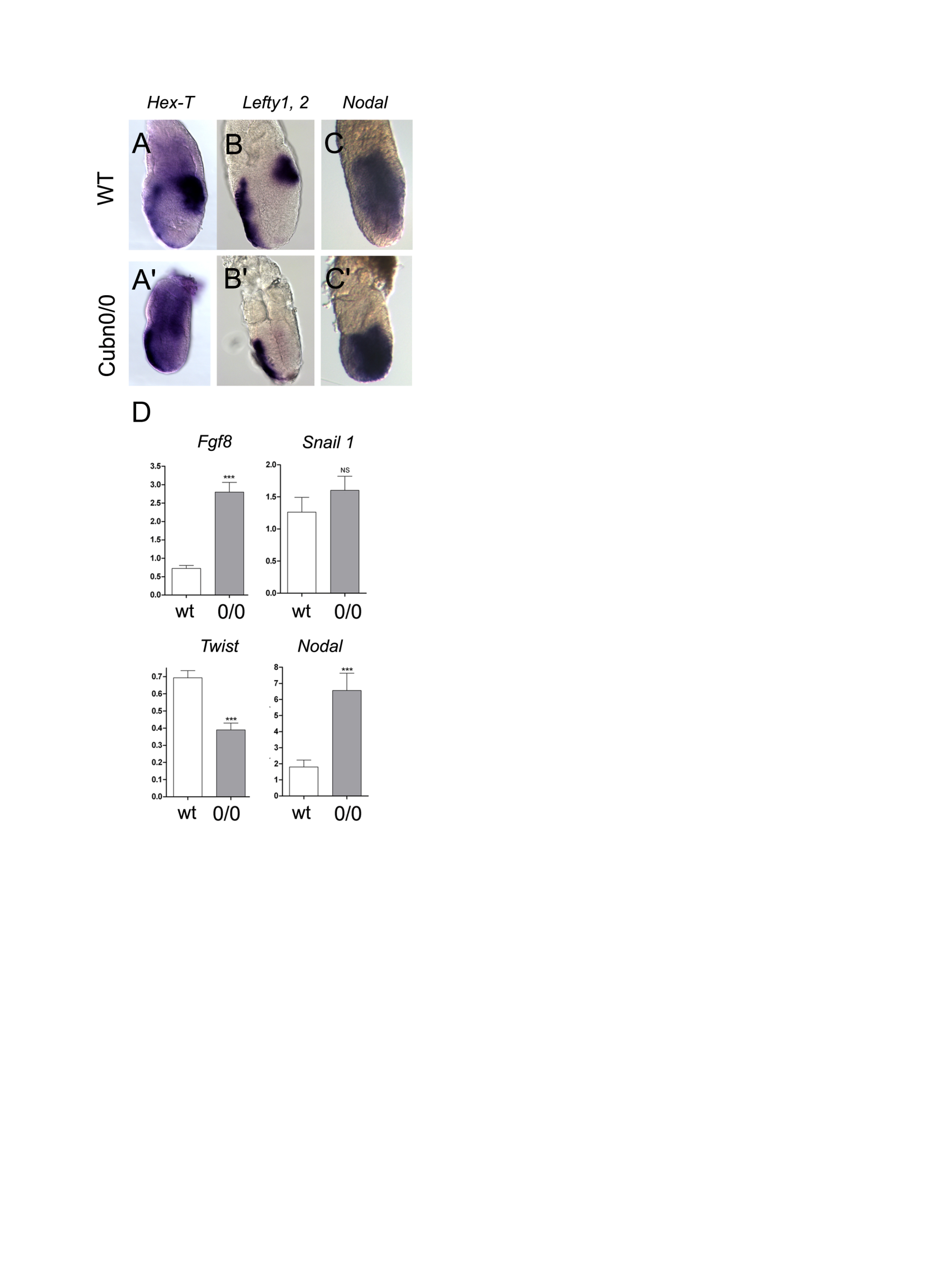


**Suppl. Figure 2.** Marker analysis of *Cubn*^0/0^ mutants at E6.5 and E7.5. (**A, B**) By E6.5, *Hex* and *Lefty-1* delineate the anterior visceral endoderm (AVE) whereas *T* and *Lefty-2* are expressed in the nascent primitive streak. (**A’, B’**) In *Cubn^0/0^* embryos *Hex* and *Lefty-1* are expressed in the anterior visceral endoderm whereas *T* expression is greatly down regulated and *Lefty-2* expression is absent. (**C**) *Nodal* is expressed in the epiblast with higher posterior expression. (**C’**) In *Cubn^0/0^* embryos, *Nodal* is homogeneously distributed in the epiblast.

(**D)** Quantitative RT-PCR performed in five mutants and seven controls for each gene analyzed at E7.5 confirms the upregulation of *Fgf8* and unmodified expression of *Snail* in mutants. The *Fgf8* target and posterior mesoderm marker *Twist* is downregulated whereas *Nodal* is strongly upregulated in the *Cubn^0/0^* embryos.


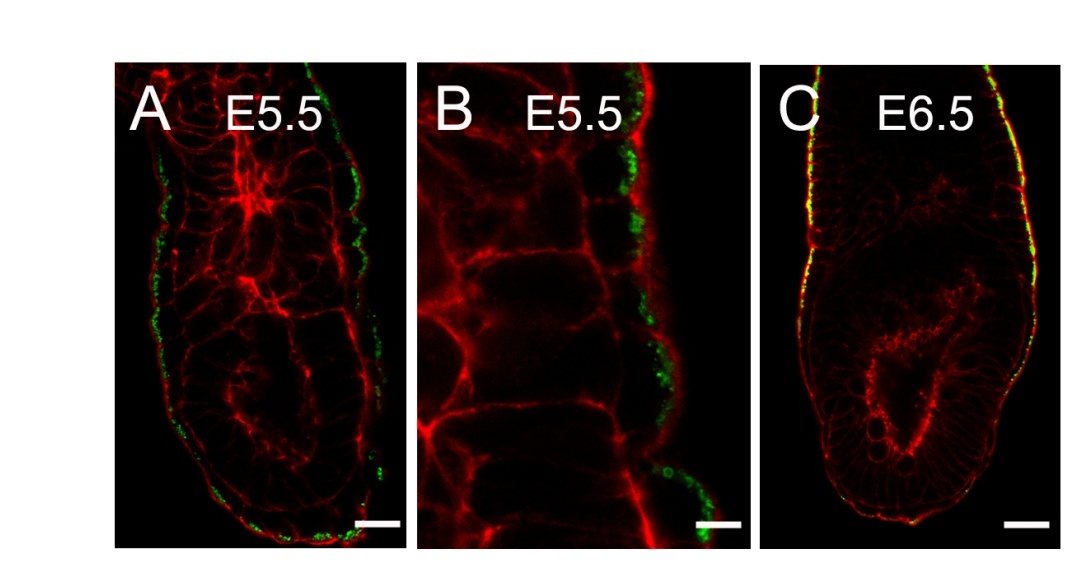


**Suppl. Figure 3.** Confocal imaging of transferrin uptake (green) and actin staining (red). (**A**) By E5.5, transferrin uptake occurs in embryonic visceral endoderm (emVE) and extraembryonic visceral endoderm (exVE) cells. (**B**) Transferrin accumulates in subapical vesicles. (**C**) By E6.5, transferrin uptake is localized mainly in exVE cells. Some uptake is also seen in emVE cells. All images show single confocal sections. Scale bars: 25 μm in A; 10 μm in B; 40 μm in C.


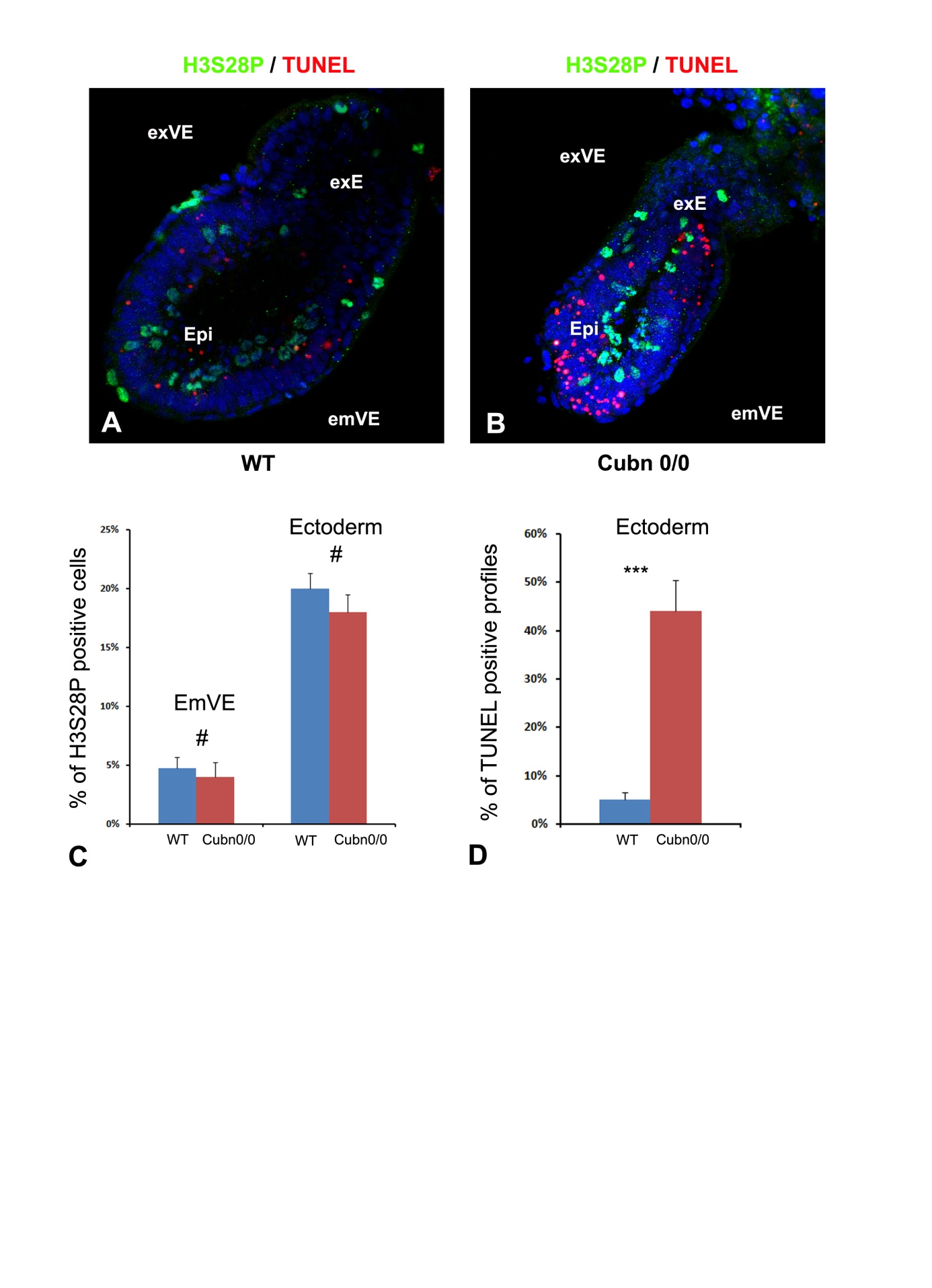


**Suppl. Figure 4.** Proliferation and TUNEL assay in E6.5 *Cubn* null embryos. (**A**, **B**) H3S28P immunofluorescence (green) and TUNEL staining (red) in a confocal section of E6.5 embryos. (**A**) H3S28P positive cells and TUNEL positive profiles are evenly scattered along the ectoderm (epiblast (Epi) and extraembryonic ectoderm(exE)) of wild-type controls. (**B**) In *Cubn* null embryos, numerous TUNEL positive profiles are concentrated in the distal anterior epiblast and the posterior extraembryonic ectoderm. (**C**) Graph showing the percentages of embryonic visceral endoderm (emVE) and ectoderm (epiblast and extraembryonic ectoderm)-specific cells positive for H3S28P-immunofluorescence in seven consecutive confocal sections of wild-type littermate (blue plot; n = 3) and *Cubn* null embryos (red plot; n =3). All results are calculated as mean and SEM. The differences between wild-type littermate and *Cubn* null embryos are not significant (emVe p-value 0.85; ectoderm p-value 0.37). (**D**) Graph showing the percentages of ectoderm (epiblast and extraembryonic ectoderm)-specific profiles for TUNEL staining in seven consecutive confocal sections of wild-type littermate (blue plot; n = 3) and *Cubn* null embryos (red plot; n =3). The difference between wild-type littermate and *Cubn* null embryos is highly significant (p-value 5.4 x 10^-4^).


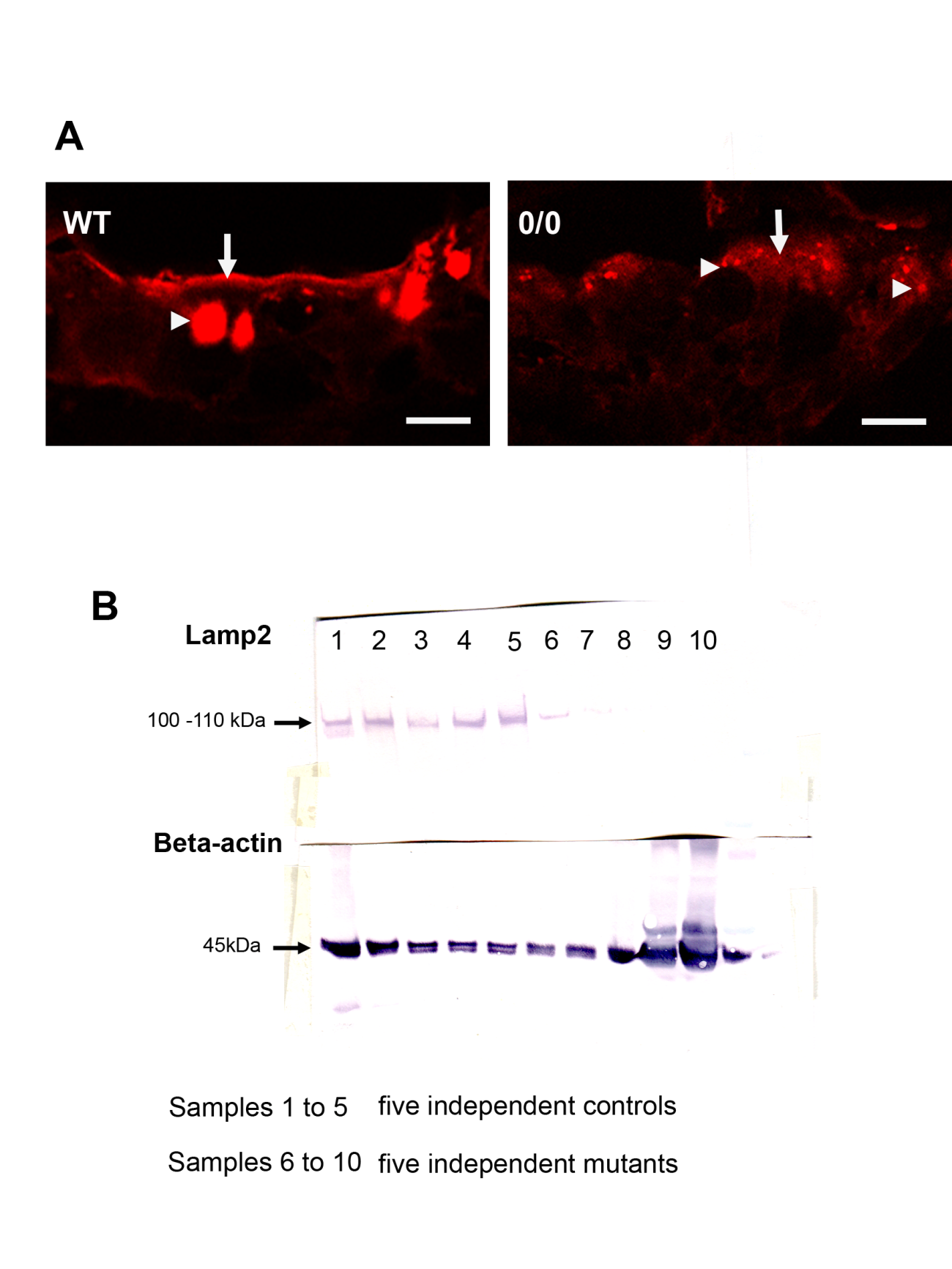


**Suppl. Figure 5**: (**A**) Clathrin adaptator complex 1 gamma-adaptin (AP1G1, AP-1) immunoreactivity is found at the plasma membrane (arrow) and large vacuoles (arrowhead). In *Cubn^0/0^* embryos, AP-1 is absent from the plasma membrane and condenses in small vesicles near the plasma membrane (arrowheads). (**B**) Full length blots showing the presence of Lamp2 and beta-actin in ten independent samples (extraembryonic visceral endoderm extracts of gastrulating control (1-5) and mutant (6-10) embryos). Scale bars: 15μm.

**Table S1.** **List of primers used in genotyping and real time RT-PCR**

| Gene | Forward Primer | Reverse Primer | Fragment | sequence |
| --- | --- | --- | --- | --- |
| *Genotyping* | | |  |  |
| *Cubilin* | 5’ATGCTCAGCTGGGACTGTCT3’ | 5’AAGGGACTAGGGACAGGTACATC3’ | 27435-27743 | NM001081084 |
|  | 5’GTTGTCAGAACCGATGAGGA3’ | 5’AAGGGACTAGGGACAGGTACATC3’ | 24181-27743* | “ “ |
| *Real time RT-PCR* | | |  |  |
| *mAmn* | 5’ CCCTACTGCCTCAAGGACAAT 3’ | 5’ GGTACCGCTCCAGGTCAAAT 3’ | 736-822 | NM033603 |
| *mBMP4* | 5’ GGCTGGAATGATTGGATTGTG 3’ | 5’ CAATGGCATGGTTGGTTGAGT 3’ | 1389-1497 | NM087554 |
| *mCubn* | 5’ ACGAGCTCCTGGACTGGTTAT 3’ | 5’ GCTCTGGTAGGCAAAGGTTC 3’ | 1438-1530 | NM_001081084 |
| *maFP* | 5’ TCCCTCATCCTCCTGCTACAT 3’ | 5’ TCTTCTCCGTCACGCACTG 3’ | 89-191 | NM007423 |
| *mFGF8* | 5’ CGCTCCGCGCTGAGCTG 3’ | 5’ CGAGTGTCAGCTGGCTTC 3’ | 31-130 | NM010205 |
| *mGATA4* | 5’ ACTCCAGCAATGCCACTAGC 3’ | 5’ TGTGCCCATAGTGAGATGACA 3’ | 1650-1739 | NM008092 |
| *mHNF4* | 5’ CCATCATCTTCTTTGATCCAG 3’ | 5’ CTCACTTGCACCTGTGACC 3’ | 989-1070 | NM008261 |
| *mHPRT* | 5’ TGGTGAAAAGGATCTCTCGAA 3’ | 5’ TCAAGGGCATATTCAACAATA 3’ | 637-727 | NM013556 |
| *nodal* | 5’ CTGTAGAGAGGGGCGGATG 3’ | 5’GGTTGAAGTCCACCTGGAACT 3’ | 695-793 | NM013611 |
| *mSnail1* | 5’ TGAAGAGATACCAGTGCCAGG 3’ | 5’ CGGAGCAGCCAGACTCTTG 3’ | 761-844 | NM011127 |
| *mTTR* | 5’ GGCTTCCCTTCGACTCTTC 3’ | 5’ ATCCAGGACTTTGACCATCAG 3’ | 29-140 | NM013697 |
| *mTwist* | 5’ CTCGGACAAGCTGAGCAAGA 3’ | 5’ CTGCAGGACCTGGTACAGGAA 3’ | 736-811 | NM011658 |
| *mvHNF1* | 5’ ATAGCTCCAACCAGACGCACA 3’ | 5’ TAGCGCACTCCTGACATCT 3’ | 1134-1236 | NM009330 |
| *mWnt3a* | 5’ GCACTCAGCCCATTCTCTGT 3’ | 5’ TCCACGTAGTTCCTGCAGAAG 3’ | 235-310 | NM009522 |
